# ﻿New record and diet of a poorly known frog, *Amolops daorum* (Amphibia, Anura) from Vietnam

**DOI:** 10.3897/zookeys.1262.172081

**Published:** 2025-12-05

**Authors:** Anh Van Pham, Cuong The Pham, Truong Quang Nguyen, Hoa Thanh Thi Nguyen, Thuong Thanh Hoang, Minh Duc Le, Thomas Ziegler

**Affiliations:** 1 Faculty of Environmental Sciences, University of Science, Vietnam National University, Hanoi, 334 Nguyen Trai Road, Hanoi 11416, Vietnam; 2 Institute of Biology, Vietnam Academy of Science and Technology, 18 Hoang Quoc Viet Road, Hanoi 10072, Vietnam; 3 Graduate University of Science and Technology, Vietnam Academy of Science and Technology, 18 Hoang Quoc Viet Road, Hanoi 10072, Vietnam; 4 Tay Bac University, Son La Province, Vietnam; 5 Central Institute for Natural Resources and Environmental Studies, Vietnam National University, Hanoi, 19 Le Thanh Tong, Hanoi 11021, Vietnam; 6 Department of Herpetology, American Museum of Natural History, Central Park West at 79; 7 th; 8 Street, New York, New York 10024, USA; 9 AG Zoologischer Garten Köln, Riehler Straße 173, D-50735 Köln, Germany; 10 Institute of Zoology, University of Cologne, Zülpicher Street 47b, D-50674 Cologne, Germany

**Keywords:** Coleoptera, distribution, Hymenoptera, morphology, prey items, stomach contents, Son La Province

## Abstract

As a result of our field surveys, a new population of the Dao Frog, *Amolops daorum* (Bain, Lathrop, Murphy, Orlov & Ho, 2003), was found in Son La Province, northern Vietnam, and its identification was based on molecular and morphological analyses. To date, knowledge about the natural history of this species is scarce, including data on its dietary ecology. Using the stomach-flushing method, we analyzed stomach contents of 132 individuals (81 males and 51 females) of *A. daorum*. We found 501 prey items (458 invertebrates, one vertebrate, and 15 unidentified items), belonging to 11 insect orders (Blattodea, Coleoptera, Dermaptera, Diptera, Hemiptera, Hymenoptera, Isoptera, Lepidoptera, Odonata, Orthoptera, Plecoptera), insect larvae, Araneae, Opiliones, Mollusca, Polydesmida, Scolopendromorpha, and a frog. The dominant prey items of the species were Coleoptera (19.16%), Hymenoptera (17.76%), insect larvae (16.57%), Hemiptera (9.98%), Araneae (7.98%), Diptera (6.99), and Orthoptera (5.79%). The importance index for these categories ranged from 5.61% to 18.45%. Hymenoptera and Coleoptera were the categories with the highest frequency of prey items, found in 55 and 54 stomachs, respectively. There was an overlap of 82.83% in the diet between males and females. Coleoptera, insect larvae, Hymenoptera, Araneae, and Orthoptera represented the most important prey categories for both sexes.

## ﻿Introduction

Globally, one-third of all amphibian species are threatened with extinction and almost half are experiencing population declines (Stuart et al. 2004; IUCN 2025). However, information on the diet ecology of many frogs is lacking. Knowledge about the diet ecology is crucial for a better understanding of the life history, population fluctuations, and the impact of habitat modification on populations of frogs (Toft 1980; Strüssmann et al. 1984; Donnelly 1991; Ogoanah and Uchedike 2011; Caldart et al. 2012; Merlita and Olga 2013). In addition, identifying the prey taxa for each species helps to clarify the impact of frogs on invertebrate control (Nakamura and Tominaga 2021). The main factors influencing the feeding ecology of frogs are food abundance, prey availability in the environment, size constraints, ecological tolerances, and climatic conditions (Schoener 1974; Toft 1980; Strüssmann et al. 1984; [Bibr B9][Bibr B21]; Pham et al. 2019a, 2023a, 2024a, b), so frogs in tropical and subtropical regions have more diverse diets than in temperate regions (Ugarte et al. 2007; Brito et al. 2013; Ngo et al. 2014). Previous studies have shown that mostly invertebrates, and sometimes small vertebrates, make up the diets of frogs, of which terrestrial and aquatic insects have been reported as preferred frog prey (Duellman and Trueb 1994; Wells 2007; Strüssmann et al. 1984; Miranda et al. 2006; Merlita and Olga 2013; Ngo et al. 2014; Pham et al. 2019a, 2024a).

The genus *Amolops* Cope, 1865 currently contains 89 species that inhabit forest streams (Frost 2025). Despite a high number of species richness, dietary studies were only focused on *Amolops larutensis* (Boulenger) and *Amolops shihaitaoi* Wang, Li, Du, Hou & Yu. In Son La Province, studies on the diet of amphibians have been conducted in several species, for example *Microhyla butleri*, *M. heymonsi* in Son La City and Phong Lai Commune; *Limnonectes bannaensis* in Copia, Muong La, and Ta Xua nature reserves (NRs); *L. nguyenorum* in Quynh Nhai and Muong Do communes; *Nanorana yunnanensis* and *Odorrana chapaensis* in Muong La Nature Reserve (NR); *Odorrana jingdongensis* in Copia and Muong La NRs; and *Polypedates megacephalus* in Muong Do and Phong Lai communes (Pham and Nguyen 2018; Pham et al. 2019a, b, 2022, 2023a, 2024a, b). Those studies demonstrated that these frogs have varied diets, with ants, beetles, dipterans, and insect larvae, representing the predominant prey.

The Dao Frog, *Amolops daorum* (Bain, Lathrop, Murphy, Orlov & Ho, 2003), was described from Vietnam, with the holotype deriving from Lao Cai Province. At present, the species is known from Lao Cai (Vietnam), Yunnan Province and Hong Kong (China), and Houaphan (Laos) (Frost 2025). This species is closely associated with streams in mixed secondary evergreen forest of larger and medium-sized hardwoods, shrubs, and arrowroot (Bain et al. 2003).

As a result of our recent field work in Muong La and Ta Xua NRs, northern Vietnam, *A. daorum* is recorded for the first time from Son La Province. In addition, we provide novel data on dietary ecology of the species by the stomach-flushing method.

## ﻿Material and methods

### ﻿Sampling

Three field surveys were conducted in Son La Province, northern Vietnam (Suppl. material 1: fig. S1): (1) Ngoc Chien Commune, within Muong La NR by Anh Van Pham and Nenh Ba Sung in October 2016; (2) Xim Vang Commune, within Ta Xua NR by Quyen The Bui and Trieu Van Dau in May 2017; (3) Hang Dong Commune, within Ta Xua NR by Anh Van Pham and Nenh Ba Sung in May 2019. Frogs were collected by hand between 20:30 and 23:00 h following the guidelines approved by the American Society of Ichthyologists and Herpetologists for animal care (Beaupre et al. 2004). In this study, we used the stomach-flushing technique to obtain stomach contents without sacrificing them (Griffiths 1986; Leclerc and Courtois 1993; Solé et al. 2005). Spatula, forceps, two syringes with thread (60 ml), and the infusion tube of soft material (silicon) were used to collect prey items in the stomach of frogs, in particular for small individuals to avoid perforations of the oesophagus and stomach. Each specimen was stomach-flushed only once following the guidelines approved by the American Society of Ichthyologists and Herpetologists for animal care (Beaupre et al. 2004). The water for flushing was taken from the streams where the frogs were captured and used after filtration. After stomach-flushing, frogs were monitored for vigour and body conditions and released within 30 min at the place of capture. Prey items were preserved in 70% ethanol. Frogs were subsequently released at the collecting site after measurements of snout–vent length (SVL), mouth width (MW) with digital calipers to the nearest 0.01 mm taken and body mass (BM) with an electronic balance. In total, 132 frogs, including 81 males and 51 females.

For taxonomic identification, two individuals were collected for voucher specimens. After having been photographed in life, animals were anesthetized and euthanized in a closed vessel with a piece of cotton wool containing ethyl acetate (Simmons 2002), fixed in 85% ethanol and subsequently stored in 70% ethanol. Specimens were deposited in the collections of the Faculty of Environmental Sciences, University of Science, Vietnam National University.

### ﻿Morphological characters

Measurements were taken with digital calipers to the nearest 0.1 mm. The following abbreviations were used (after Pham et al. 2023b):

**SVL** snout–vent length (from the back of mandible to tip of snout);

**HL** head length (from the back of mandible to tip of snout);

**MW** maximum head width (across angles of jaws);

**SE** distance from tip of snout to anterior corner of eye;

**SND** distance from nostril to the tip of snout;

**END** eye to nostril distance (distance from anterior corner of eye to the nostril);

**ED** eye diameter;

**TD** tympanum diameter;

**FLL** forelimb length (from tip of disc of finger III to axilla);

**HLL** hindlimb length (from tip of disc of fourth toe to groin);

**FL** femur length (from vent to knee);

**TL** tibia length (from knee to tarsus).

### ﻿Molecular data

We sequenced two new samples of *Amolops daorum* collected from Son La Province. We used the protocols of Le et al. (2006) for DNA extraction, amplification, and sequencing. A fragment of the mitochondrial gene, 16S ribosomal RNA, of approximately 550 bp was amplified and sequenced using the primer pair LR N 13398 (5′-CGCCTGTTTACCAAAAACAT-3′; forward), LR J 12887 (5′-CCGGTCTGAACTCAGATCACGT-3′; reverse) (Simon et al. 1994). Sequences were compared with those from GenBank using Basic Local Alignment Search Tool (BLAST) searches.

### ﻿Stomach content analysis

Prey items were identified under a microscope (Olympus SZ 700) based on identification keys (i.e. Naumann et al. 1991; Thai 2003; Johnson and Triplehorn 2005; Brusca et al. 2016). The maximum length (*L*) and width (*W*) of each prey item were measured to the nearest 0.1 mm using either calipers or a calibrated ocular micrometer fitted to a microscope. The volume (*V*) of prey items was calculated using the formula for a prolate spheroid (*π* = 3.14, Pham et al. 2024a: *V* = 4π / 3 × (*L* / 2) × (*W* / 2) 2 (mm^3^). The index of relative importance (*IRI*) was used to determine the importance of each food category. This index provides a more informed estimation of prey item consumption than any of the three components alone by using the following formula: *IRI* = (%*F* + %*N* + %*V*) / 3 (Caldart et al. 2012), where *F* is the frequency of prey occurrence in stomachs and *N* is the total number of prey items concerning all prey items. We used the reciprocal Simpson’s heterogeneity index, 1-*D*, to calculate dietary heterogeneity: *D* = ∑[*n*_i_(*n*_i_ – 1)] / [*N* (*N* – 1)]. Where ni is the number of prey items in the i^th^ taxon category and *N* is the total number of prey items (Krebs 1999).

To estimate prey evenness, we used Shannon’s index of evenness. Evenness is calculated from the equation: *J*′ = *H*′ / Hmax = *H*′ / ln *S*. The maximum diversity (Hmax) that could occur is that which would be found in a situation in which all taxa had equal abundance (H′ = Hmax = ln S), S is the total number of prey taxa, and *H*′ is the Shannon–Weiner index of taxon diversity. The value of *H*′ is calculated from the equation: *H*′ = –∑(*P*_i_ × ln *P*_i_), where the quantity *P*_i_ is the proportion of total prey items belonging to the ith taxon for the total prey items of the sample (Magurran 2004; Muñoz-Pedreros and Merino 2014).

The diet overlap between males and females was calculated by measuring the percentage overlap (Krebs 1999; Caldart et al. 2012), defined by the following equation:

$P_{\mathrm{jk}}=\left[\sum^{n}\left({\mathrm{minimumP}}_{\mathrm{ij}},P_{\mathrm{ik}}\right)\right]100$

Where *P*_jk_ is the percentage overlap between groups j and k, *P*_ij_ and *P*_ik_, respectively, represent the proportions of prey i consumed by groups j and k, and n is the total number of prey categories.

Statistical analyses were performed with the SPSS 20.0 (SPSS Inc., Chicago, Illinois, USA) and with the significance level set to *p* < 0.05 for all analyses. Data are presented as mean ± standard deviation (SD) unless otherwise noted. To evaluate the relationships between the frog SVL and the prey volume of each individual, we calculated the following index values including minimum, maximum, mean prey item volume, and total prey volume (Nakamura and Tominaga 2021). Kendall’s tau-b statistics were used to examine relationships between the frog SVL and the minimum, maximum, mean prey item volume, and total prey volume (Nakamura and Tominaga 2021). Wilcoxon’s rank sum test (*W*) was used to examine the size of prey items, the number of prey items, and prey volume from frogs of different sexes. Multiple regression analysis was used to examine the relationships between body size (SVL and MW), prey width, and prey volume.

## ﻿Results

### ﻿New record of *Amolops daorum* from Son La Province

Two sequences (GenBank accession numbers PX499615 (IB A.6422), PX499616 (IB A.6423) of *Amolops* specimens from Son La Province were almost identical (99.58% and 99.60% similar, respectively) to the sequence available in GenBank of *A. daorum* (accession number FJ417151 from the type locality, Hoang Lien National Park, Lao Cai Province).

### ﻿Morphological description of two collected specimens

**Figure 1.**
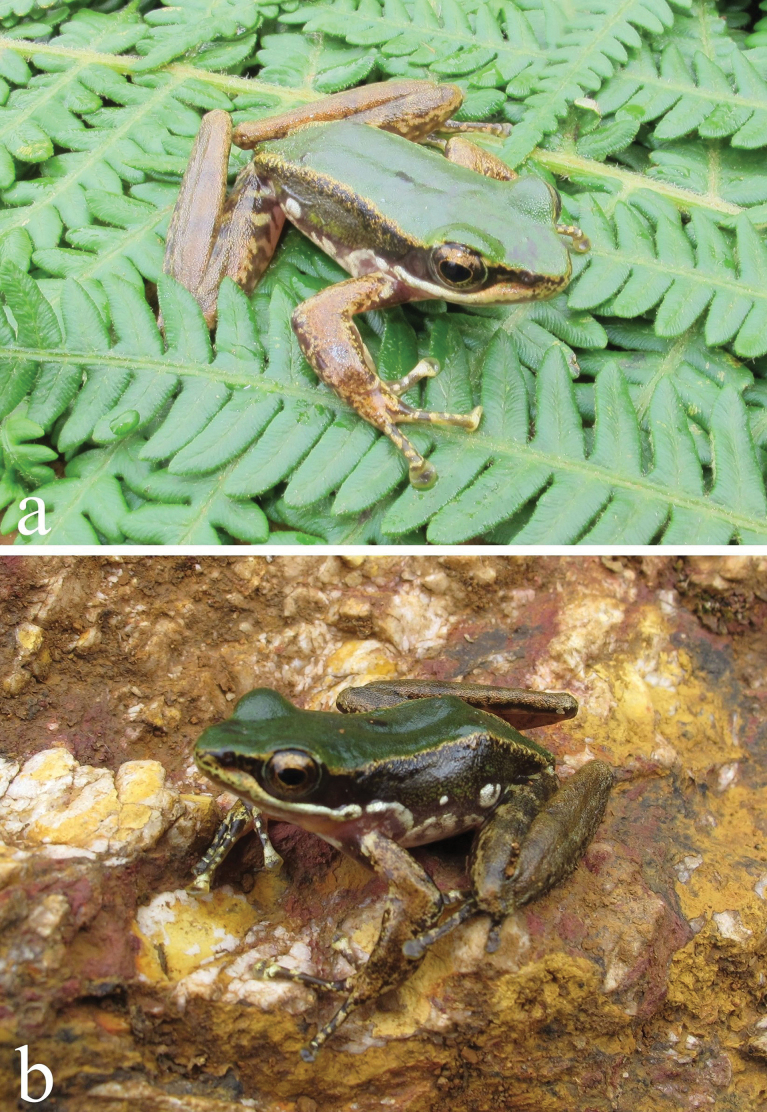
*Amolops daorum* in Son La Province, Vietnam. a. Adult male; b. Adult female.

Morphological characteristics of the specimens collected in Son La Province resemble the diagnosis of *Amolops daorum* (Fig. 1) (Bain et al. 2003): SVL 36.8 mm in the male and 49.9 mm in the female; head large, broad and flat (HL/SVL 0.35 in the male and 0.36 in the female, MW/SVL 0.31 in the male and 0.32 in the female), longer than wide (HL 12.6 mm in the males and 17.8 in the female; MW 11.1 mm in the male and 16.0 mm in the female); snout pointed anteriorly in dorsal view; nostril lateral, closer to snout tip than to eye (SND 2.6 mm in the male and 3.5 mm in the female, END 2.9 mm in the male and 3.7 mm in the female); canthus rostralis distinct; loreal region concave; snout length greater than eye diameter (SE 5.5 mm in the male and 7.5 mm in the female; ED 5.2 mm in the male and 6.1 mm in the female); eyes very large, pupil horizontally oval; tympanum distinct, round (TD/ED 0.40 in the male and 0.43 in the female); vomerine teeth absent; tongue cordiform, notched posteriorly; vocal sac opening on floor of mouth at corner.

Forelimb long (FLL/SVL 0.72 in the male and 0.68 in the female); relative finger lengths I<II<IV<III; fingers without webbing; tips of fingers expanded into discs, second to fourth with circummarginal grooves; tip of first finger smaller, without circummarginal groove; subarticular tubercles oval, formula 1, 1, 2, 2; metatarsal tubercle indistinct; glandular nuptial pad on finger I.

Hindlimb very long (HLL/SVL 1.94 in the male and 2.07 in the female); tibia longer than thigh (FL 19.9 mm in the male and 28.8 mm in the female; TL 22.1 mm in the male and 32.1 mm in the female); relative toe length I<II<III<V<IV; tips of toes expanded into discs; width of disc of toe IV smaller than that of finger III; subarticular tubercles oval, formula 1, 1, 2, 3, 2; inner metatarsal tubercle elongate; outer metatarsal tubercle absent.

Skin texture in life: dorsal surface and lateral side of head and body smooth, except few very small tubercles present on vent; supratympanic fold indistinct; dorsolateral fold distinct, from rear of upper eyelid to near vent; ventral surface smooth.

#### ﻿Colouration in life

Dorsal surface of head and dorsum green; dorsolateral stripe yellow; lateral side of head and tympanum dark brown; flank greyish brown with white glandular spot; upper part of iris golden; a white stripe extending from the tip of the snout along upper lip to the anterior joint of the shoulder on each side, more distinct posteriorly; dorsal surface of fore and hind limbs light grey; throat, chest and belly white; ventral surface of fore and hind limbs cream; toe webbing brown (Fig. 1).

#### ﻿Measurements and weight of the individual’s stomach-flushing

SVL min–max: 29.5–38.0 mm; mean ± SD: 35.19 ± 2.07 mm, *n* = 81), MW 9.5–12.6 mm (11.07 ± 0.68 mm, *n* = 81), and body mass (BM 1.63–7.42 g, 3.58 ± 0.85 g, *n* = 81) in males and SVL 37.5–53.6 mm (44.68 ± 4.74 mm, *n* = 51); MW 10.8–17.0 mm (14.11 ± 1.68 mm, *n* = 51); BM 3.42–16.89 g, 8.25 ± 3.27 mm, *n* = 51) in females. There were strong positive correlations between the morphological measurements (SVL and MW: *r* = 0.962, *p* < 0.001; SVL and BM: *r* = 0.915; *p* < 0.001; MW and BM: *r* = 0.897, *p* < 0.001) (Suppl. material 1: fig. S2).

#### ﻿Ecological notes

Frogs in all sites were observed at night between 20:30 and 23:00, on trees nearby a stream, about 0.2–1.0 m above the ground, at elevations between 1,800 and 2,200 m a.s.l. The surrounding habitat was evergreen forest of large and, medium-sized hardwoods. The air temperature was 20–28 °C and the relative humidity was 70–85%.

#### ﻿Distribution

In Vietnam, this species was recorded from Lao Cai and Son La provinces (Frost 2025 and this study). Elsewhere, this species is known from Yunnan Province and Hong Kong (China) and Houaphan (Laos Province) (Frost 2025).

### ﻿Food items and prey dimension

A total of 132 individuals (81 males and 51 females) of *A. daorum* were captured in Son La Province for stomach flushing. We found 501 prey items (310 items in 81 males, 191 items in 51 females) in stomachs of frogs. The number of prey items per individual was 1.0–23.0 (mean: 3.8 items, *n* = 501). The median number of preys among individuals with food in their stomachs was one (3.83) for males and one (3.75) for females with no statistically significant inter sexual difference (Wilcoxon’s rank sum test, *W* = 5335.500, *P* = 0.809) (Suppl. material 1: fig. S3). The maximum number of preyfound in a single stomach was 23 for males and 11 for females.

Mean prey-item length was 7.81 mm (min–max: 1.0–35.0 mm, *n* = 501); mean prey-item width was 2.48 mm (0.5–10.0 mm, *n* = 501). The average dietary volume per item was 56.64 mm^3^ (min–max 0.13–1674.67 mm^3^, *n* = 501).

The median measurements of 310 prey items from male stomachs were as follows: length 6.62 mm (range: 1.0–33.0 mm), width 1.97 mm (range: 0.5–10.0 mm), and volume 28.45 mm^3^ (range: 0.13–680.33 mm^3^) (Table 1).

**Table 1.** Summary (Total, Mean, and range) of the prey item number (*N*), width (*W*, in mm), length (*L*, in mm), and volume (*V*, in mm^3^) data for males and females.

	Prey item	
	Male (*n* =310)	Female (*n* = 191)
*W*	0.50–10.00	0.70–10.00
*L*	1.00–33.00	1.50–35.00
*V*_total	1.57–988.29	36.85–848.06
*V*_minimum	0.13–392.50	0.51–261.67
*V*_maximum	1.57–680.33	3.66–1674.67
*V*_mean	1.26–392.50	2.14–480.55
*N*	1.00–11.00	4.00–35.00

The measurements for the 191 items from female stomachs were as follows: length 9.76 mm (range: 1.5–35.0 mm), 3.31 mm (range: 0.7–10.0 mm), and volume 102.38 mm^3^ (range: 0.51–1674.67 mm^3^).

The length, width and volume of prey items were significantly different in between females and males (Wilcoxon’s rank sum test; length: *W* = 67944, *P* < 0.001; width: *W* = 63786, *P* < 0.001; volume: *W* = 63770, *P* < 0.001). The median volume per stomach content was 214.96 mm^3^ (min–max 1.57–1922.2 mm^3^, *n* = 132); the median volume per stomach content in males was 108.9 mm^3^ (range: 1.57–988.29 mm^3^, *n* = 81) and in females 383.41mm^3^ (range: 4.28–1922.20 mm^3^, *n* = 51) (Fig. 3), with a statistically significant intersexual difference (Wilcoxon’s rank sum test, *W* = 4410.5, *P* < 0.001) (Table 2).

**Table 2.** Results of analysis Wilcoxon’s rank sum test (*W*) examining the impact sex on the number of prey items in stomachs, volume of prey, length of prey items, and width of prey items of *Amolops daorum*.

Variables	Source	*W*	*P*
Number of prey items in stomachs	Sex	5335.5	0.809
Volume of prey in stomachs	Sex	4410.5	<0.001
Length of prey items	Sex	67944	<0.001
Width of prey items	Sex	63786	<0.001
Volume of prey items	Sex	6377	<0.001

**Figure 2.**
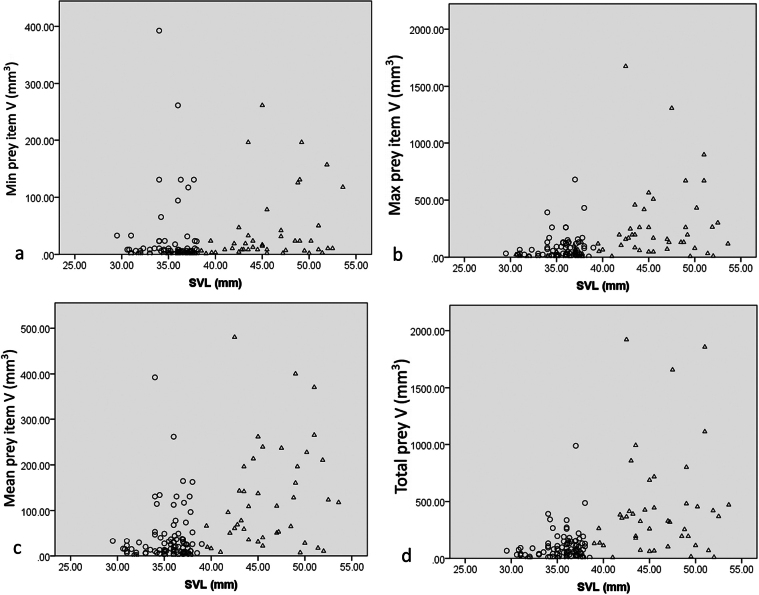
Relationships between the frog SVL and a. The minimum; b. Maximum; c. The mean prey item volume; d. Total prey volume. Dots: Females; Open triangles: Males.

There was a positive correlation between frog SVL and the minimum, mean, maximum prey volume, and the total prey volume (Kendall’s tau-b, minimum: tau = 0.198, *P* = 0.002, mean: tau = 0.343, *P* < 0.001, maximum: tau = 0.308, *P* < 0.001, the total prey volume: tau=0.354, *P* < 0.001) (Table 3, Fig. 2).

**Table 3.** Results of analysis Kendall’s tau-b examining relationships between the frog SVL and the minimum, maximum, mean prey item volume, and total prey volume.

Variables	Source	Tau-b	*P*
Minimum prey volume	SVL	0.198	0.002
Mean prey volume	SVL	0.343	<0.001
Maximum prey volume	SVL	0.308	<0.001
The total prey volume	SVL	0.354	<0.001

**Figure 3.**
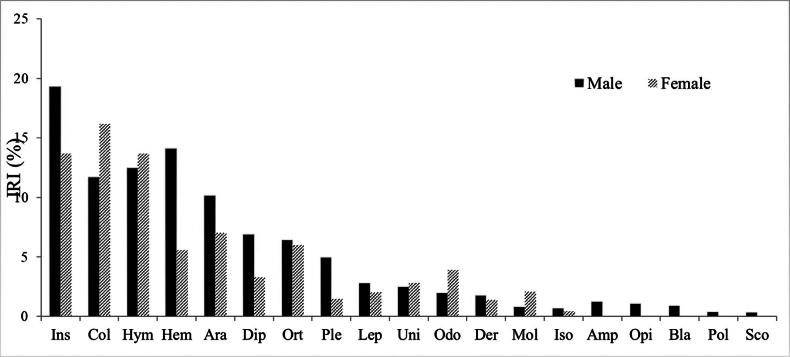
Importance index (*IRI*) for prey categories consumed by males (black) vs females (cross) of *Amolops daorum* in Vietnam. Coleoptera (Col), insect larvae (Ins), Araneae (Ara) Lepidoptera (Lep), Orthoptera (Ort), Hymenoptera (Hym), Polydesmida (Pol), Blattodea (Bla), Dermaptera (Der), Unidentified (Uni), Mollusca (Mol), Hemiptera (Hem), Plecoptera (Ple), Diptera (Dip), Opiliones (Opi), Scorpiones (Sco), Scolopendromorpha (Scol), Odonata (Odo), Amphibia (Amp).

The result of multiple regression analysis indicated that both SVL and MW were associated positively with prey length, width, and volume (prey length: *F*_2,499_ = 25.110, *P* < 0.001, *R*^2^ = 0.092 and the regression equation is W = 0.499SVL − 0.360MW + ε; prey width: *F*_2,499_ = 56.719, *P* < 0.001, *R*^2^ = 0.186, and the regression equation is W = 0.085SVL + 0.153MW + ε; prey volume: *F*_2,499_ = 28.58, *P* < 0.005, *R*^2^ = 0.103, and the regression equation is V = 6.931SVL + 4.042MW + ε) because there was a significant relationship between SVL and MW (SVL and MW: *r* = 0.962, *p* < 0.001).

### ﻿Dietary diversity

We identified 11 insect orders (Blattodea, Coleoptera, Dermaptera, Diptera, Hemiptera, Hymenoptera, Isoptera, Lepidoptera, Odonata, Orthoptera, Plecoptera), insect larvae, Araneae, Opiliones, Mollusca, Polydesmida, Scolopendromorpha, and a frog (Table 2). The highest frequency of occurrence (%F) of prey items identified was Hymenoptera (16.18%), followed by Coleoptera (15.88%), insect larvae (14.71%), Araneae (10.29%), Hemiptera (9.71%), Orthoptera (7.06%), and Diptera (6.47) while the highest proportion (%N) prey items was Coleoptera (19.16%), followed by Hymenoptera (17.76%), insect larvae (16.57%), Hemiptera (9.98%), Araneae (7.98%), Diptera (6.99), and Orthoptera (5.79%). The highest volume of occurrence (%V) of prey items identified was insect larvae (24.07%), followed by Coleoptera (14.41%), Hymenoptera (12.19%), Hemiptera (10.44%), Orthoptera (8.49%), Araneae (8.32%), and Odonata (7.63%). In the comparisons by the IRI (%), insect larvae (18.45%), followed by Coleoptera (16.48%), Hymenoptera (15.38%), Hemiptera (10.04%), Araneae (8.87%), Orthoptera (7.11%), and Diptera (5.61%) were found to be the important prey groups of the species (Table 4).

**Table 4.** Dietary composition (%) of *Amolops daorum*: frequency of occurrence (*F*), numeric proportion (*N*), volume proportion (*V*) and overall importance (*IRI*) value of each prey taxon (*n* = 501 prey items).

	*F*	%*F*	*N*	%*N*	*V*	%*V*	*IRI*
Mollusca	5	1.47	6	1.20	704.93	2.48	1.72
Araneae	35	10.29	40	7.98	2361.80	8.32	8.87
Opiliones	3	0.88	3	0.60	63.43	0.22	0.57
Polydesmida	1	0.29	1	0.20	33.27	0.12	0.20
Scolopendromorpha	1	0.29	1	0.20	18.84	0.07	0.19
Blattodea	2	0.59	2	0.40	94.20	0.33	0.44
Coleoptera	54	15.88	96	19.16	4088.02	14.41	16.48
Dermaptera	7	2.06	7	1.40	424.47	1.50	1.65
Diptera	22	6.47	35	6.99	961.23	3.39	5.61
Hemiptera	33	9.71	50	9.98	2961.61	10.44	10.04
Hymenoptera	55	16.18	89	17.76	3457.53	12.19	15.38
Isoptera	3	0.88	3	0.60	110.95	0.39	0.62
Lepidoptera	8	2.35	11	2.20	690.28	2.43	2.33
Odonata	6	1.76	6	1.20	2164.25	7.63	3.53
Orthoptera	24	7.06	29	5.79	2408.91	8.49	7.11
Plecoptera	15	4.41	23	4.59	405.71	1.43	3.48
Insecta larva	50	14.71	83	16.57	6830.05	24.07	18.45
Amphibia	1	0.29	1	0.20	261.67	0.92	0.47
Unidentified	15	4.41	15	2.99	333.63	1.18	2.86

The total dietary breadth of *A. daorum* from Vietnam was 0.88 (Simpson’s index of diversity) and Shannon’s evenness was 1.0. The diversity index of prey categories of adult males (0.88 with an evenness index of 1.02) was higher than that of adult females (0.87 with an evenness index of 0.97) (Table 5). There was an overlap of 82.83% in the diet of males and females. Coleoptera, insect larvae, Hymenoptera, Araneae, Hemiptera, and Orthoptera represented the important (*IRI* > 5) prey categories for both sexes (Suppl. material 2, Fig. 3).

**Table 5.** Simpson’s Index of Diversity and Shannon’s Evenness among sexes in the diet of *Amolops daorum* from Vietnam.

Contents	Simpson’s index 1-*D*	Shannon’s evenness *E*
Males	0.88	1.00
Females	0.88	1.01
Total	0.87	0.97

## ﻿Discussion

The Dao Frog was described from Lao Cai Province, Vietnam in 2003 as Rana (Odorrana) daorum. Stuart (2005) provided specific localities of *Rana daorum* for Laos, and later Bain et al. (2006) provided a record from Huaphan Province, Laos. Stuart (2008) regarded the species as a member of *Amolops*. In the *Amolops monticola* group, Wu et al. (2020) discussed molecular phylogenetics and provided a new record from Yunnan Province, China. Poyarkov et al. (2021) suggested, without noting why, that the Laos record required confirmation. Stuart et al. (2025) summarized literature, taxonomy, habitat, and detailed range for Laos, and suggested that the Lao populations require taxonomic reevaluation.

The diet of *A. daorum* is primarily composed of beetles, spiders, crickets, grasshoppers, ants and other invertebrate groups which is similar to food content of many other frogs (Brasileiro et al. 2010; Caldart et al. 2012; Ngo et al. 2014; Pham et al. 2019a, 2023a, 2024a). The terrestrial prey clearly indicate the general habitat use by *A. daorum* (Pham et al. 2019a, 2024a). Besides prey categories as insects, *A. daorum* also consumes other invertebrates, such as snails, caterpillars, cockroaches, termites. Remarkably, an adult frog of the genus *Leptobrachella*, measured of 5.0 mm in width and 20.0 mm in length was found in the stomach of a female of *A. daorum*, approximately 0.92% of the total prey volume of the sampled individuals. Most adult amphibians consume a wide range of small invertebrate prey items (Wells 2007). Among some instances, frogs eating frogs are often the subject of anecdotal observations (Toledo et al. 2007). The snout–vent length was significantly related to prey volume in *A. daorum*, and this is consistent with the scale-efficiency hypothesis (Forsman 1996). The prey volume of *A. daorum* in females was larger than that in males because females with larger body sizes, so they likely consume larger prey items than males (Pham et al. 2023a). We also found a slight difference in the dietary composition of males versus females in *A. daorum*. Amphibia, Opiliones, Blattodea, Polydesmida, and Scolopendromorpha were detected exclusively in the diet of males. Despite the differences in diets between males and females, Coleoptera, insect larvae, Hymenoptera, Araneae, and Orthoptera represented the important prey categories for both sexes.

Pham and Nguyen (2018) and Pham et al. (2019a, 2023a, 2024a, b) reported the dietary composition of *Nanorana yunnanensis* (Anderson), *Limnonectes bannaensis* Ye, Fei, Xie & Jiang, *Odorrana chapaensis* (Bourret) and *O. jingdongensis* Fei, Ye & Li, four other frog species also occurring in Copia, Muong La, and Ta Xua NRs in Son La Province, Vietnam. *Amolops daorum*, *Nanorana yunnanensis*, *Limnonectes bannaensis*, *O. chapaensis* and *O. jingdongensis* inhabit similar environmental conditions, i.e. near streams with mixed evergreen forests of larger and medium-sized hardwoods and shrubs. There is a significant difference between the diet composition of *A. daorum* and those from the sympatric frogs; however, Coleoptera constitutes the most important prey category for these aforementioned species. The important (IRI > 5%) preys in the diet of *A. daorum* include insect larvae (18.45%), Coleoptera (16.48%), Hymenoptera (15.38%), Hemiptera (10.04%), Araneae (8.87%), Orthoptera (7.11%), and Diptera (5.61%), while the diet of *N. yunnanensis* (*n* = 45) primarily consists of Coleoptera (21.28%), insect larvae (15.56%), Lepidoptera (13,08%), Mollusca (8.04%), and Hemiptera (6.46%) (Pham and Nguyen 2018). Regarding *Limnonectes bannaensis*, Pham et al. (2024b) reported that the diets of three populations (*n* = 157) were composed predominantly of Coleoptera (38.04%), Hymenoptera (9.31%), Araneae (7.93%), Polydesmida (7.07%), Orthoptera (6.37%), and Lepidoptera (5.01%). With regard to *Odorrana chapaensis*, the diets of one population (*n* = 73) mostly comprise Coleoptera (32.45%), Lepidoptera (9.57%), Blattodea (7.99%), Orthoptera (7.12%), and Hymenoptera (6.08%) (Pham et al. 2019a). As for *Odorrana jingdongensis*, the food content of three populations (*n* = 159) mainly consists of Coleoptera (32.0%), insect larvae (11.1%), Araneae (9.06%), Orthoptera (7.49%), Hymenoptera (6.33%), and Lepidoptera (6.25%) (Pham et al. 2023b). The predominant consumption of Coleoptera is one of the reasons for the high trophic niche overlap between the species. Also, species may be foraging in similar places, considering that species that share the same habitat tend to have a similar diet (Duellman and Trueb 1994; Guidali et al. 2000; Sabagh et al. 2010). Compositional Analysis pointed out that differently from other species, *N. yunnanensis* exhibited a considerable level of selectivity in its diet, preying on insect larvae, Lepidoptera, Mollusca, and Hemiptera more frequently than their expected availability in the habitat. This result brings some light to the relevance of studying syntopic species to elucidate feeding adaptations.

Because the number of individuals analyzed in this study is small, it is difficult to support comparisons of the diet by sites. Nonetheless, our preliminary results show that the diets of *Amolops daorum* Muong La NR and Ta Xua NR are different in terms of number of prey items ingested and prey size. In addition, frog individuals in Ta Xua had a broader trophic spectrum than those in Muong La.

## ﻿Additional information

### Use of AI

No use of AI was reported.

### Author ORCIDs

Anh Van Pham https://orcid.org/0000-0002-6023-3418

Cuong The Pham https://orcid.org/0000-0001-5158-4526

Truong Quang Nguyen https://orcid.org/0000-0002-6601-0880

Hoa Thanh Thi Nguyen https://orcid.org/0009-0005-3504-4286

Thuong Thanh Hoang https://orcid.org/0009-0006-5758-3165

Minh Duc Le https://orcid.org/0000-0002-2953-2815

Thomas Ziegler https://orcid.org/0000-0002-4797-609X

### Data availability

All of the data that support the findings of this study are available in the main text or Supplementary Information.

## Supplementary materials

Supplementary material 1 Supplementary figures Data type (measurement/occurrence/multimedia/etc.) Data type: doc Explanation note **fig. S1.** Maps showing the previously recorded location (black circle) and the new localities: (1) Ngoc Chien Commune; (2) Xim Vang Commune; (3) Hang Dong Commune in Vietnam. **fig. S2.** Dispersion diagrams from Pearson’s correlations between (a) snout-vent length and mouth width, (b) snout-vent length and body mass, and (c) mouth width and body mass of *Amolops daprrum* in Son La Province, Vietnam. **fig. S3.** Boxplots representing factors that differed significantly among treatments: a) number of prey items of Amolops daorum per stomach and sex; b) Prey volume per stomach and sex. https://binary.pensoft.net/file/1485286This dataset is made available under the Open Database License (http://opendatacommons.org/licenses/odbl/1.0/). The Open Database License (ODbL) is a license agreement intended to allow users to freely share, modify, and use this Dataset while maintaining this same freedom for others, provided that the original source and author(s) are credited.Anh Van Pham, Cuong The Pham, Truong Quang Nguyen, Hoa Thanh Thi Nguyen, Thuong Thanh Hoang, Minh Duc Le, Thomas Ziegler

Supplementary material 2 Dietary composition (%) of *A. daorum* Data type doc Explanation note Frequency of occurrence, numeric proportion, volume proportion, and overall importance value of each prey taxon for males and females. https://binary.pensoft.net/file/1485287This dataset is made available under the Open Database License (http://opendatacommons.org/licenses/odbl/1.0/). The Open Database License (ODbL) is a license agreement intended to allow users to freely share, modify, and use this Dataset while maintaining this same freedom for others, provided that the original source and author(s) are credited.Anh Van Pham, Cuong The Pham, Truong Quang Nguyen, Hoa Thanh Thi Nguyen, Thuong Thanh Hoang, Minh Duc Le, Thomas Ziegler
