# Supplementary material for: ﻿New record and diet of a poorly known frog, Amolops daorum (Amphibia, Anura) from Vietnam
Source: Zookeys. 2025 Dec 5;1262:203–19. doi: 10.3897/zookeys.1262.172081 (PMC12701358; doi:10.3897/zookeys.1262.172081)
Supplement: Supplementary material 1 — Supplementary figures [file zookeys-1262-203_article-172081__-s001.doc]

**New record and diet of a poorly known frog, *Amolops daorum* (Amphibia: Anura) from Vietnam**


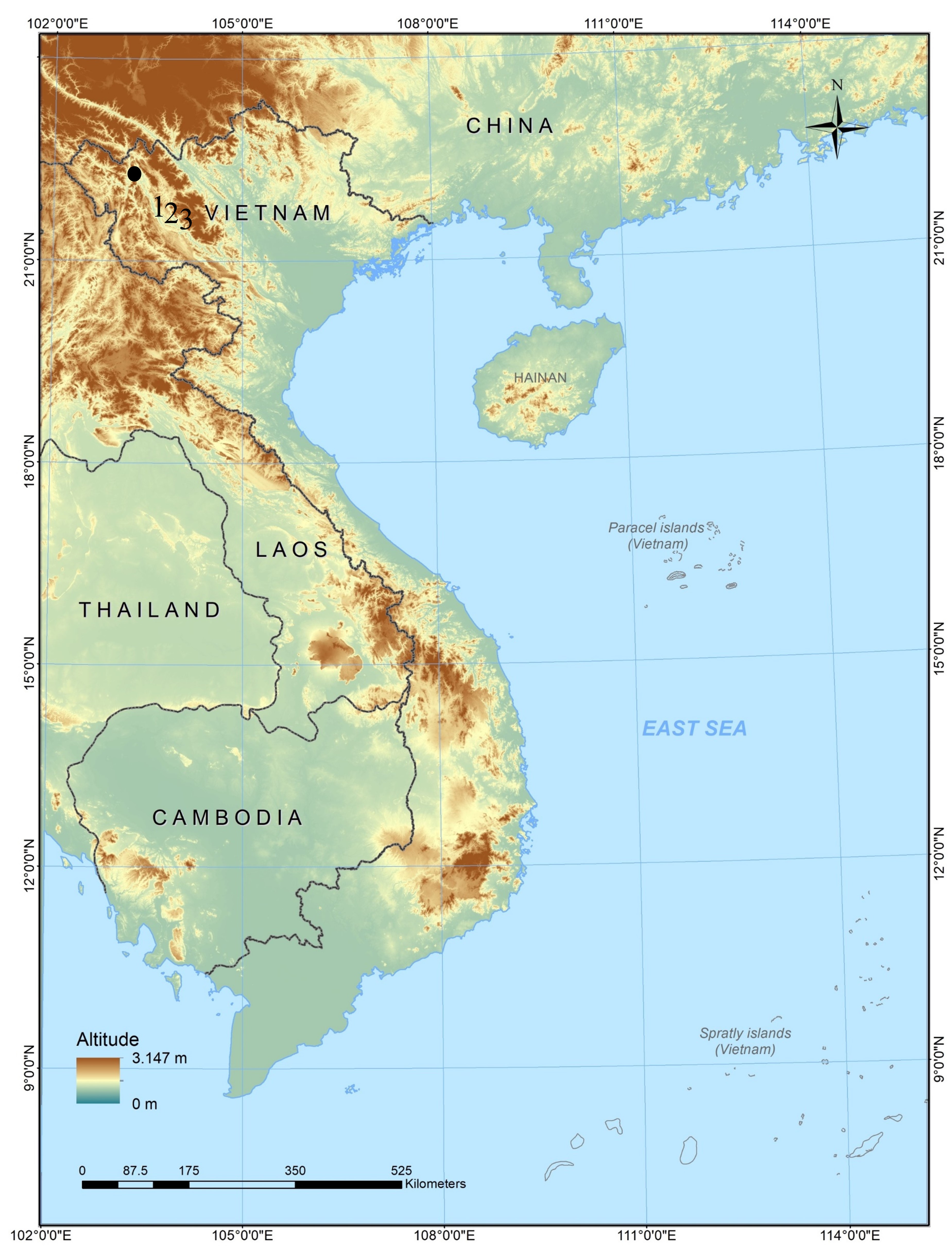


**Figure 1.** Maps showing the previously recorded location (black circle) and the new localities: (1) Ngoc Chien Commune; (2) Xim Vang Commune; (3) Hang Dong Commune in Vietnam


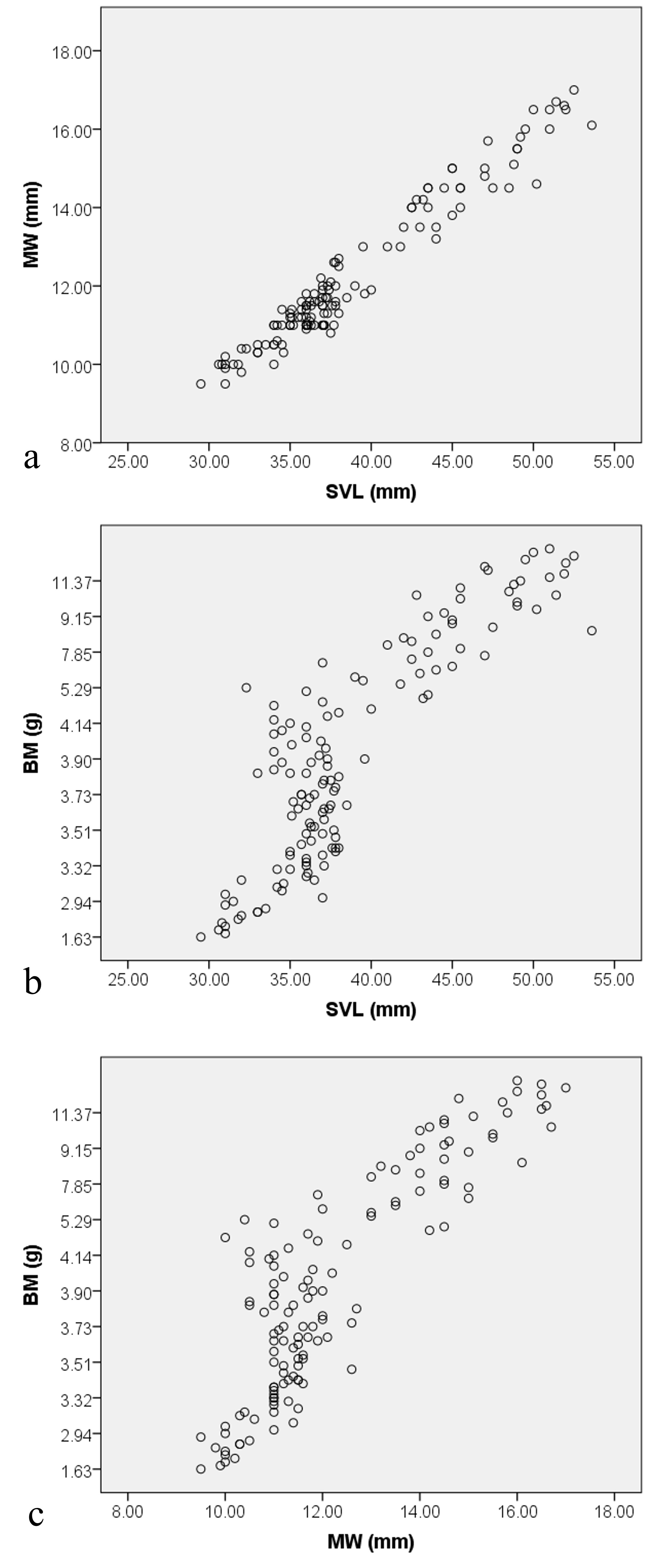


**Figure 2.** Dispersion diagrams from Pearson’s correlations between (a) snout-vent length and mouth width, (b) snout-vent length and body mass, and (c) mouth width and body mass of *Amolops daprrum* in Son La Province, Vietnam


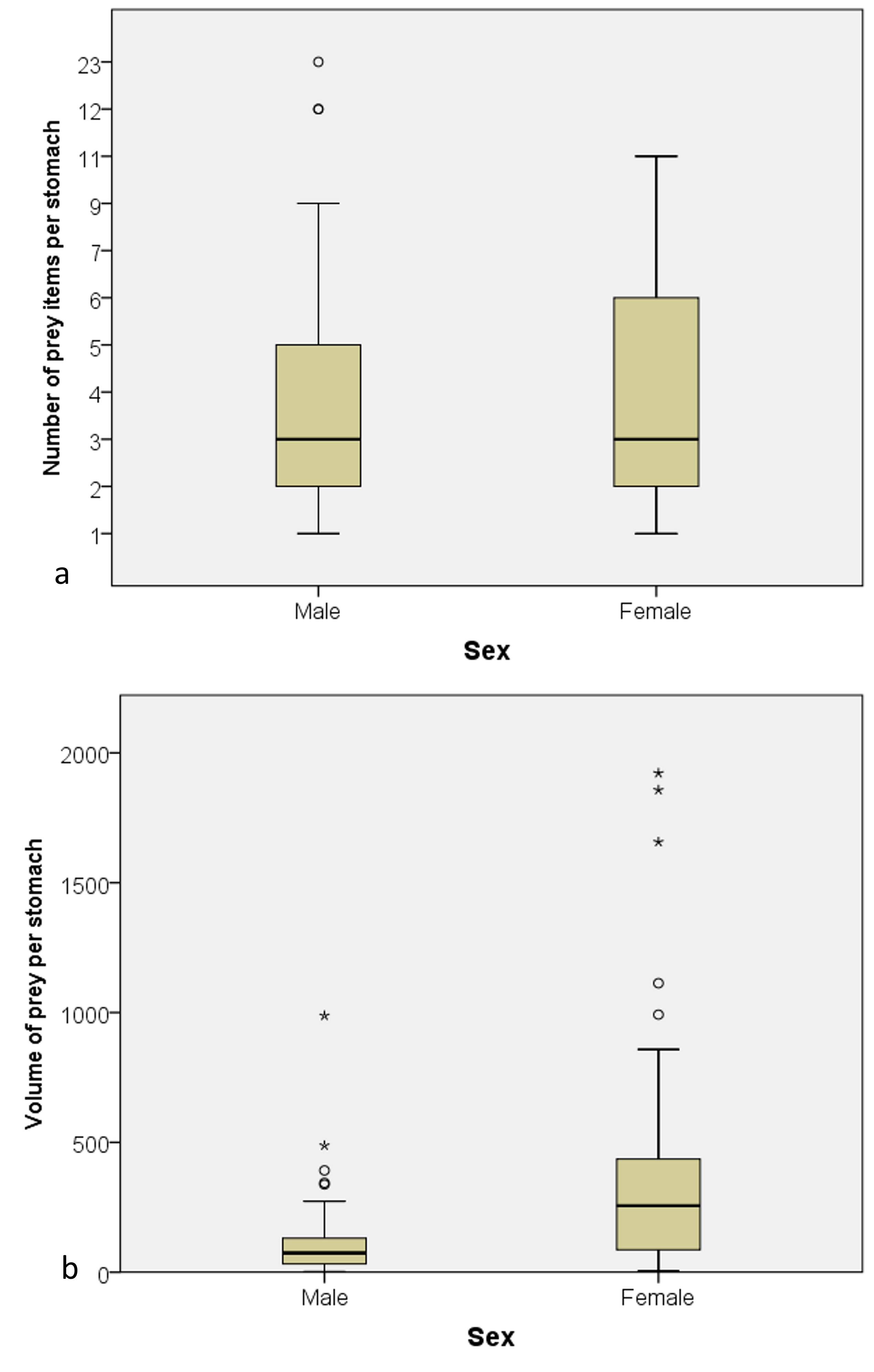


**Figure 3.** Boxplots representing factors that differed significantly among treatments: a) number of prey items of *Amolops daorum* per stomach and sex; b) Prey volume per stomach and sex
