# Supplementary material for: ﻿New record and diet of a poorly known frog, Amolops daorum (Amphibia, Anura) from Vietnam
Source: Zookeys. 2025 Dec 5;1262:203–19. doi: 10.3897/zookeys.1262.172081 (PMC12701358; doi:10.3897/zookeys.1262.172081)
Supplement: Supplementary material 2 — Dietary composition (%) of A. daorum [file zookeys-1262-203_article-172081__-s002.doc]

**New record and diet of a poorly known frog, *Amolops daorum* (Amphibia: Anura) from Vietnam**

**Table 1.** Dietary composition (%) of *A. daorum:* frequency of occurrence, numeric proportion, volume proportion, and overall importance value of each prey taxon for males and females

|  | F | | %F | | N | | %N | | V | | %V | | IRI | |
| --- | --- | --- | --- | --- | --- | --- | --- | --- | --- | --- | --- | --- | --- | --- |
|  | male | female | male | female | male | female | male | female | male | female | male | female | male | female |
| Mullusca | 2 | 3 | 0.99 | 2.19 | 2 | 4 | 0.66 | 0.80 | 71.70 | 633.23 | 0.81 | 3.24 | 0.82 | 2.08 |
| Araneae | 19 | 16 | 9.36 | 11.68 | 22 | 18 | 7.21 | 3.59 | 1223.03 | 1138.77 | 13.87 | 5.82 | 10.15 | 7.03 |
| Opiliones | 3 | - | 1.48 | - | 3 | - | 0.98 | - | 63.43 | - | 0.72 | - | 1.06 | - |
| Polydesmida | 1 | - | 0.49 | - | 1 | - | 0.33 | - | 33.27 | - | 0.38 | - | 0.40 | - |
| Scolopendromorpha | 1 | - | 0.49 | - | 1 | - | 0.33 | - | 18.84 | - | 0.21 | - | 0.34 | - |
| Blattodea | 2 | - | 0.99 | - | 2 | - | 0.66 | - | 94.20 | - | 1.07 | - | 0.90 | - |
| Coleoptera | 26 | 29 | 12.81 | 21.17 | 52 | 44 | 17.05 | 8.78 | 464.17 | 3623.85 | 5.26 | 18.53 | 11.71 | 16.16 |
| Dermaptera | 4 | 3 | 1.97 | 2.19 | 4 | 3 | 1.31 | 0.60 | 172.74 | 251.72 | 1.96 | 1.29 | 1.75 | 1.36 |
| Diptera | 15 | 7 | 7.39 | 5.11 | 23 | 12 | 7.54 | 2.40 | 512.87 | 448.37 | 5.81 | 2.29 | 6.91 | 3.27 |
| Hemiptera | 23 | 10 | 11.33 | 7.30 | 34 | 16 | 11.15 | 3.19 | 1754.01 | 1207.59 | 19.89 | 6.18 | 14.12 | 5.56 |
| Hymenoptera | 31 | 25 | 15.27 | 18.25 | 52 | 37 | 17.05 | 7.39 | 453.14 | 3004.39 | 5.14 | 15.36 | 12.49 | 13.67 |
| Isoptera | 2 | 1 | 0.99 | 0.73 | 2 | 1 | 0.66 | 0.20 | 43.96 | 66.99 | 0.50 | 0.34 | 0.71 | 0.42 |
| Lepidoptera | 3 | 5 | 1.48 | 3.65 | 5 | 6 | 1.64 | 1.20 | 466.81 | 223.46 | 5.29 | 1.14 | 2.80 | 2.00 |
| Odonata | 4 | 2 | 1.97 | 1.46 | 4 | 2 | 1.31 | 0.40 | 227.91 | 1936.33 | 2.58 | 9.90 | 1.96 | 3.92 |
| Orthoptera | 13 | 9 | 6.40 | 6.57 | 17 | 12 | 5.57 | 2.40 | 642.53 | 1766.38 | 7.28 | 9.03 | 6.42 | 6.00 |
| Plecoptera | 11 | 4 | 5.42 | 2.92 | 17 | 6 | 5.57 | 1.20 | 349.72 | 56.00 | 3.96 | 0.29 | 4.99 | 1.47 |
| Insecta larva | 35 | 15 | 17.24 | 10.95 | 61 | 22 | 20.00 | 4.39 | 1818.61 | 5011.44 | 20.62 | 25.63 | 19.29 | 13.66 |
| Amphibia | 1 | - | 0.49 | - | 1 | - | 0.33 | - | 261.67 | - | 2.97 | - | 1.26 | - |
| Unidentified | 7 | 8 | 3.45 | 5.84 | 7 | 8 | 2.30 | 1.60 | 148.10 | 185.52 | 1.68 | 0.95 | 2.47 | 2.79 |
